# Improving ECMO therapy: Monitoring oxygenator functionality and identifying key indicators, factors, and considerations for changeout

**DOI:** 10.1051/ject/2023047

**Published:** 2024-03-15

**Authors:** Salman Pervaiz Butt, Nabeel Razzaq, Yasir Saleem, Bill Cook, Salman Abdulaziz

**Affiliations:** 1 Perfusionist & ECMO Specialist, Heart Vascular and Thoracic Institute, Cleveland Clinic PO BOX: 112412 Abu Dhabi United Arab Emirates; 2 Perfusion Department, Cleveland Clinic PO BOX: 112412 Abu Dhabi United Arab Emirates; 3 Clinical Perfusionist, Department of CTVS, All India Institute of Medical Science Rishikesh; 4 Clinical Perfusionist, Perfusion Department, Glenfield Hospital Leicester UK; 5 Consultant of Cardiovascular Critical Care, Co-Chair of ECMO Task Force, Department of Health United Arab Emirates

**Keywords:** Extracorporeal membrane oxygenation (ECMO), Circuit Change out, Monitoring, Key Indicators

## Abstract

*Introduction*: The optimal timing for extracorporeal membrane oxygenation (ECMO) circuit change-out is crucial for the successful management of patients with severe cardiopulmonary failure. This comprehensive review examines the various factors that influence the timing of oxygenator replacement in the ECMO circuit. By considering these factors, clinicians can make informed decisions to ensure timely and effective change-out, enhancing patient outcomes and optimizing the delivery of ECMO therapy. *Methodology*: A thorough search of relevant studies on ECMO circuits and oxygenator change-out was conducted using multiple scholarly databases and relevant keywords. Studies published between 2017 and 2023 were included, resulting in 40 studies that met the inclusion criteria. *Discussion*: Thrombosis within the membrane oxygenator and its impact on dysfunction were identified as significant contributors, highlighting the importance of monitoring coagulation parameters and gas exchange. Several factors, including fibrinogen levels, pre and post-membrane blood gases, plasma-free hemoglobin, D-dimers, platelet function, flows and pressures, and anticoagulation strategy, were found to be important considerations when determining the need for an oxygenator or circuit change-out. The involvement of a multidisciplinary team and thorough preparation were also highlighted as crucial aspects of this process. *Conclusion*: In conclusion, managing circuit change-outs in ECMO therapy requires considering factors such as fibrinogen levels, blood gases, plasma-free hemoglobin, D-dimers, platelet function, flows, pressures, and anticoagulation strategy. Monitoring these parameters allows for early detection of issues, timely interventions, and optimized ECMO therapy. Standardized protocols, personalized anticoagulation approaches, and non-invasive monitoring techniques can improve the safety and effectiveness of circuit change-outs. Further research and collaboration are needed to advance ECMO management and enhance patient outcomes.

## Introduction

Extracorporeal membrane oxygenation (ECMO) is a life-saving technology used in critical care medicine to provide temporary support to patients with severe cardiac or pulmonary dysfunction. The use of ECMO has gained significant attention, especially in cases of acute respiratory distress syndrome (ARDS) and critical COVID-19 patients. Several studies have investigated the role of ECMO and its associated factors, shedding light on its efficacy and optimal management strategies.

Maintaining the optimal performance of the ECMO oxygenator is crucial for ensuring the well-being of the patient. While the components of an ECMO circuit, particularly the oxygenator, have a long lifespan, they do not last forever, and each disposable element ultimately has finite capabilities. Consequently, it becomes necessary to replace the oxygenator or the entire circuit to sustain the efficacy of ECMO therapy. The process of changing out the oxygenator or circuit is a safe and routine procedure, provided it is carefully planned and performed in accordance with established standard operating procedures to ensure the uninterrupted and effective support of patients relying on ECMO therapy. This certainly minimizes any potential risks or disruptions in patient care and ultimately optimizes patient outcomes.

When considering an ECMO oxygenator or circuit change-out, several factors need to be taken into account. These factors include circuit flows, pressures, anticoagulation levels, fibrinogen levels, pre and post-membrane blood gases, plasma-free hemoglobin, fibrinogen levels, and D-dimers.

Vigilant monitoring of circuit pressures, such as transmembrane pressure (delta P), along with anticoagulation parameters like Activated Clotting Time (ACT) and anti-Factor Xa levels, is imperative. Circuit pressure monitoring is very useful for detecting issues within the circuit, assessing blood flow resistance, and ensuring patient safety, as abnormal circuit pressures can be indicative of thrombosis. Concurrently, anticoagulation monitoring is vital to maintain the delicate balance between preventing clot formation and minimizing bleeding risks, helping to adjust anticoagulant medication dosages to safeguard patients from both thromboembolic events and hemorrhagic complications [1, 2].

The successful management of patients undergoing ECMO therapy relies on a careful evaluation of various parameters to determine the necessity of oxygenator or circuit change-outs. Critical factors to consider when making these decisions include monitoring pre and post-membrane blood gases, fibrinogen levels, and plasma-free hemoglobin. These parameters are essential in assessing the functionality and safety of the ECMO circuit, as well as detecting potential complications such as coagulopathy, gas exchange inefficiencies, and circuit damage. Furthermore, tracking markers like D-dimers and fibrinogen aids in identifying thrombus formation within the circuit. This review underscores the importance of regular monitoring and highlights the need for further research to establish standardized protocols for optimal timing in ECMO oxygenators or circuit change-outs [3, 4].

## Methodology

A thorough search of relevant studies on ECMO oxygenators and circuit change-out was conducted using multiple scholarly databases and relevant keywords. Studies published between 2017 and 2023 were included, resulting in 40 studies that met the inclusion criteria. The review process involved open-source journal publications and the Google Scholar database. Special attention was accorded to the methods of fact collection and the validity of information garnered from secondary sources. The research engagement employed all the provisions of ethical conduct in research.

## Discussion

Extracorporeal membrane oxygenation (ECMO) is a vital therapy for critically ill patients with severe respiratory or cardiac failure. However, complications like bleeding and thrombosis can negatively affect patient outcomes. This review examines recent literature to explore key factors that influence the decision to change an ECMO circuit. By understanding these factors, clinicians can make informed decisions to optimize patient care and improve ECMO outcomes.

### Fibrinogen levels

Regular monitoring of fibrinogen levels is crucial in patients undergoing ECMO to assess their coagulation status and optimize outcomes. Several studies emphasize the significance of monitoring fibrinogen levels in managing bleeding and thrombosis, as well as determining the need for circuit change-outs [3, 5, 6].

Findings from a retrospective study by Genty et al. suggest that changing the ECMO circuit in patients did not increase the risk of mortality. Replacing the ECMO circuit in patients experiencing severe and persistent bleeding can significantly reduce both clinical bleeding and the requirement for red blood cell transfusions. The study also revealed that fibrinogen levels and platelet counts, important markers of coagulation, progressively decreased before the circuit change but significantly increased afterward. This indicates that circuit changes may play a role in improving hemostasis and mitigating bleeding complications during ECMO therapy. Conversely, in patients with thrombosis, circuit changes did not have a significant impact on bleeding events or red blood cell transfusions. The study did not find any significant changes in oxygenation parameters between the pre-and post-circuit change periods. These findings emphasize the potential benefits of circuit changes in managing bleeding complications and highlight the importance of monitoring fibrinogen levels as a key factor in assessing coagulation status during ECMO support [3].

Basken et al. identified predictors of circuit exchange in patients with membrane oxygenator (MO) thrombosis. They observed significant changes in fibrinogen levels, D-dimer levels, platelet count, and heparin dose leading up to MO exchange, suggesting ongoing coagulation, consumption of coagulation factors and platelets, and inadequate anticoagulation. Fibrinogen median levels changed from 306 mg/L at day 0 before oxygenator changeout to 405 mg/L at day 3 post changeout. D dimers median changed from 1 *μg*/*mL* to 7.6*μg*/*mL* and *Platelets count was also increased after changeout* [5].

Worku et al. conducted a retrospective study involving ECMO patients and found significant reductions in fibrinogen concentration on the first day after ECMO: (3.96 g/L −2.71 g/L). Hemoglobin also decreased from (114 g/L to 85 g/L) and platelet count reduced (190 × 10^9^/L to 108 × 10^9^/L) for VA ECMO patients. Similar reductions were found in VV ECMO patients; Fibrinogen (5.41 g/L, −2.98 g/L), Hemaglobin (107 g/L to 71 g/L), and platelets (215 × 10^9^/L to 106 × 10^9^/L). A low platelet count (<50 × 10^9^/L) or fibrinogen levels of <1.8 g/L were associated with a (50%) higher likelihood of packed red blood cell transfusion [6].

Doyle et al. highlighted the activation of the coagulation system, platelet dysfunction, and altered fibrinolysis as factors contributing to both thrombosis and bleeding in ECMO patients. Understanding these hemostatic changes is essential for improving patient management [7].

Mansour et al. studied COVID-19 patients on ECMO and found that bleeding events occurred in 29% of cases, thrombotic events in 16%, and a combination of both in 20%. Longer ECMO support duration was associated with higher bleeding and thrombotic events. A fibrinogen level of >6 g/L at cannulation was associated with an elevated risk of thrombosis [8]. A level of <1.5 g/L before cannulation is associated with bleeding and mortality.

In conclusion, regular monitoring of fibrinogen levels in ECMO patients is crucial for optimizing patient outcomes, detecting abnormalities, and making informed decisions regarding circuit change-outs and anticoagulation management. Keeping Fibrinogen levels with therapeutic ranges >2 g/L and <6 g/L may be beneficial in helping prevent bleeding issues thus preventing the need to change the ECMO circuit.

### Pre and post oxygenator blood gases

Pre and post-blood gas analysis is crucial for evaluating oxygenator performance and determining the optimal time for change-out in ECMO. Clinicians assess arterial blood gas values before and after passing through the oxygenator to gauge its efficiency in removing carbon dioxide and oxygenating the blood. Monitoring these parameters enables early detection of suboptimal oxygenator function, allowing timely intervention and prevention of complications. These practices optimize ECMO therapy, enhance patient safety, and improve outcomes for critically ill individuals.

Epis et al. conducted a study on the gas exchange efficiency of the membrane lung (ML) during ECMO. It revealed that ML oxygen transfer (V’O2ML) is influenced by extracorporeal blood flow and the oxygen content difference between each ML side. ML carbon dioxide removal (V’CO2ML) depends on ML gas flow and CO_2_ concentration at the sweep gas outlet. Pseudomembranous deposits on ML fibers can hinder gas exchange, leading to a decline in V’O2ML known as the “shunt effect.” Clot formation around fibers can create a “dead space effect” that negatively impacts V’CO2ML. Monitoring these effects is crucial to recognize ML function decline. Real-time monitoring of the ECMO circuit enables timely detection of complications and improves procedural safety. Integrating a real-time monitoring system with standard measurements enhances the analysis of ECMO functioning and provides a comprehensive understanding of the ML and native lung interaction [9]. The study introduces the idea of using CO_2_ removal capacity as a marker for oxygenator aging and suggests that this can be a valuable indicator for timely oxygenator replacement. However, the paper lacks specific clinical validation and practical implementation details.

Continuous monitoring of veno-venous ECMO using the Landing® monitor provides valuable insights into the interdependency between the artificial membrane lung (ML) and the patient’s native lung (NL). The Landing monitor offers continuous measurements of ML oxygen transfer, hemoglobin levels, blood saturation, and monitors hemodynamic data, and drains negative pressure. Real-time monitoring facilitates adjustments to optimize ventilator and ECMO settings, improving performance, and potentially reducing risks and costs. Evaluating oxygen transfer aids in assessing device performance and minimizing unnecessary replacements [10].

### Plasma Free Hb

Hemolysis can occur due to infections, certain diseases, and certain medicines, but also due to mechanical issues from the ECMO circuit. Doyle et al. mention plasma free hemoglobin has been shown to occur with ECMO flow rates of >147 mL/kg/min [7]. Extreme positive or negative pressure from the ECMO, small cannula, and excessive RPMs are also a source. High levels of plasma-free hemoglobin (fHb) are cytotoxic and have secondary effects on the kidneys causing acute kidney injury. This is caused by the build-up of the pfHb structures in the kidney’s glomerular filtration apparatus. fHb scavenges nitric oxide and causes endothelial dysfunction and platelet aggregation, and minimizing this is beneficial.

The assessment of fHb levels has emerged as an important factor in evaluating hemolysis and guiding clinical decision-making in ECMO patients. Monitoring fHb levels provides insights into the occurrence and severity of hemolysis, aiding in determining when an oxygenator/circuit changeout is necessary.

Dufour et al. analyzed ECMO-associated hemolysis, highlighting the importance of laboratory assessment in managing hemolytic events. Their findings shed light on the complex nature of ECMO-associated hemolysis, optimizing patient care during ECMO therapy [11].

Studies by Pan and Omar examined the frequency of elevated fHb in adult ECMO patients and its association with circuit changes and in-hospital mortality. Both studies identified elevated fHb levels as common during adult ECMO and demonstrated associations with longer durations of support. Omar et al. found fHb levels of >50 mg/dL after 24 h showed an indication for mortality highlighting the clinical relevance of monitoring fHb levels in ECMO patients [12], however, Pan et al. found that severe hemolysis requiring circuit changes was uncommon, were found almost exclusively on VV ECMO and were non statistically significant with in-hospital mortality [13].

Valladolid et al. focused on the role of fHb in promoting thrombosis within the ECMO circuit. They highlighted the interaction between fHb and plasma von Willebrand factor, emphasizing the need for further research to investigate this interaction and develop strategies for reducing thrombotic risks during ECMO therapy [14].

Hazboun et al. identified increased fHb levels as an early indicator of neonatal ECMO circuit impairment. Mean fHb was taken 48 h prior and increased from 26.45 mg/dL to 76 mg/dL, a cause of this could be as oxygenator delta pressure rises the rpm is increased to maintain flows, and this rise in mechanical stress causes more fHb. Monitoring fHb, along with other parameters, can aid in the timely detection of circuit deterioration and appropriate interventions in neonatal ECMO cases [15].

Neal et al. did a study looking at pfHb levels in Cardiohelp, CentriMag, and PediMag circuits where 5 out of 146 patients had a pfHb spike over 100 mg/dL. One of these was CentriMag bivads which was treated with a pump reseating, one PediMag complete ECMO circuit was changed, and the rest were Cardiohelp and were changed. All of this resulted in a reduction of pfHb after 12 h or less to at least a third of the peak levels. If this drop is not seen it suggests the cause of pfHb has not been addressed and care should be taken while using CentriMags as the cause of pfHb could be the pump itself rather than the oxygenator. This does not pose a problem for Cardiohelp as it’s integrated and so both are changed simultaneously regardless of the cause [16].

In contrast, a study by Appelt et al. demonstrates that hemolysis was prevalent in VA ECMO patients, however, this is caused by different indications and not the ECMO itself [17].

Collectively, these studies enhance our understanding of fHb and possible reasons for a rise in ECMO therapy. Monitoring fHb levels provides valuable insights into hemolysis occurrence and severity. While not an indication for changing a circuit on its own, pHb levels provide a good picture of hemolysis and add to the decision-making process regarding ECMO oxygenator/circuit change out. Further research is needed to validate and expand upon these findings, improving outcomes in ECMO settings.

### D-Dimers

Monitoring D-dimer (DD) levels can provide valuable information regarding the presence of clot formation within the ECMO oxygenator. Elevated DD levels suggest ongoing activation of the coagulation system and the breakdown of fibrin clots. While increased DD levels are not specific to clotting within the oxygenator, they serve as a marker of overall coagulation activity.

The retrospective study by Lubnow et al. emphasized the importance of early identification of oxygenator dysfunction during prolonged ECMO use. DD levels raised from 15 (9–20) to 30 (21–35) mg/dL within 3 days before exchange and declined significantly within 1 day thereafter to 13 (7–17) mg/dL. The rise in plasma D-dimer concentration, in the absence of other factors, was identified as a helpful predictor for membrane oxygenator (MO) exchange in heparin-coated ECMO systems. Monitoring DD levels can facilitate timely exchanges and prevent sudden loss of function due to clot formation [4].

Similarly, Dornia et al. investigated the relationship between elevated D-dimer levels and thrombotic clots in the MO during VV ECMO. The study highlighted the significance of D-dimer testing in predicting clot formation and preventing system-induced coagulation disorders. Persistent high D-dimer levels (11.5 [6.5–15.5] mg/L to 35.0 [34, 35] mg/L) for >2 days correlated with increased clot volume detected by multidetector computed tomography (MDCT). Levels then dropped following membrane exchange where D-dimer levels decreased significantly 12 [8–16] mg/L. This underscores the importance of monitoring MOs during ECMO therapy to prevent critical situations [18].

Aqsa Shakoor conducted a retrospective study focusing on D-dimer levels in COVID-19 patients requiring VV-ECMO support. Elevated D-dimer levels (>3,000 ng/mL) were associated with a shorter time from admission to cannulation and a longer duration of ECMO support. The consistent decrease in D-dimer values after circuit exchanges indicated the presence of a thrombus within the oxygenator. Elevated D-dimer levels were proposed as indicators of increased disease severity in COVID-19 and predictors of a longer ECMO course. The paper highlights the complexity of interpreting D-dimer in ECMO-supported COVID-19 patients. Weaknesses include the small sample size, retrospective design, and limited statistical significance in some findings, making further research necessary for definitive conclusions [19].

These studies collectively highlight the utility of monitoring D-dimer levels in assessing clot formation and oxygenator dysfunction during ECMO therapy. Early detection of elevated D-dimer levels in addition to other tests can prompt timely interventions, such as MO exchange, to optimize patient care and prevent complications. Further research is warranted to validate these findings and explore additional markers for evaluating coagulation disorders and disease severity in ECMO patients.

### Platelet levels

Platelet function plays a significant role in oxygenator change-out during ECMO management. Impaired platelet function can lead to increased clot formation within the oxygenator, compromising its performance. Monitoring platelet function helps identify suboptimal oxygenator function and guide the timing of oxygenator/circuit change-out. By ensuring adequate platelet function, the risk of clot formation can be minimized, optimizing ECMO therapy and improving patient outcomes.

Balle et al. observed decreased platelet counts and reduced platelet aggregation in adult patients on ECMO. On day 1 only, decreased platelet activation was found, this did not continue from day 1 to 3. This suggests potential platelet consumption or activation within the ECMO circuit. However, when considering platelet aggregation relative to platelet count, it was similar to healthy controls. In addition, bound Fibrinogen levels increased indicating that platelet function may not be universally impaired during ECMO [20].

A study on platelet transfusions in children undergoing ECMO found that overall, platelet transfusions did not significantly affect oxygenator function as measured by postoxy O_2_ levels and differences in pre and post membrane pressures. However, in patients with the lowest pre-transfusion oxygenator function, platelet transfusions were associated with worsened oxygenator function. This highlights the need for careful consideration of platelet transfusion strategies and anticoagulation management, especially in patients with lower oxygenator function [21].

A systematic review identified a significant knowledge gap regarding platelet function during ECMO in adult patients. However, reduced expression of platelet adhesion receptors decreased platelet activation markers and reduced platelet aggregation were reported in the included studies. Bleeding episodes were frequent, while thromboembolic events were not reported, emphasizing the need to investigate the associations between platelet function and bleeding/thromboembolic complications during ECMO. The study limitation mentions that the patient cohorts were heterogenous with respect to underlying disease which could affect platelet count. With this in mind, there may also be a strong association between platelet count and platelet aggregation as mentioned by Balle et al. previously [22].

Winnersbach et al. more recently found that reducing platelet count in an ECMO system limited platelet activation and the formation of neutrophil extracellular trap (NET). This resulted in decreased clot stability. However, clot formation within the circuit still occurred, indicating the significant role of platelets in clot formation despite reduced count [23].

Analyzing membrane oxygenators (MOs) from ECMO patients, another study revealed that some MOs exhibited high von Willebrand factor (vWF) loading, particularly near the gas fiber crossing points. Patients with coagulation abnormalities showed highly loaded MOs, suggesting increased levels of thrombogenic vWF multimers and their accumulation during ECMO therapy. The study has limitations as It’s an in vitro study, so its relevance to clinical situations is limited. The small sample size (*n* = 5) raises concerns about generalizability and residual platelets in the platelet-poor group could confound results. The focus on local anticoagulation strategies lacks direct testing in this context. While the study offers insights, its limitations and the gap between lab and real-world scenarios should be considered [24].

In summary, these studies provide insights into the complex relationship between platelet function, clot formation, and ECMO support. Monitoring platelet count is crucial during ECMO treatment, and measuring aggregation, and activation may also be useful. Further research is necessary to optimize management strategies, including platelet transfusions, anticoagulation, and the prevention of bleeding and thromboembolic events in ECMO patients.

### Flows and pressures

The role of flow and pressures in assessing oxygenator function during ECMO therapy has been investigated in several studies. Zakhary et al. emphasized the importance of monitoring membrane lung (ML) dysfunction, highlighting various factors that can contribute to ML dysfunction and the need for prompt recognition. Pressure monitoring across the ML and measuring oxygen transfer were discussed as monitoring methods, providing guidelines for ML replacement based on abnormalities and inadequate gas exchange [25].

Sarathy et al. explored the use of flow measurement as an alternative method to detect obstructions in the ECMO circuit, specifically focusing on oxygenator thrombosis. Their results demonstrated that higher levels of oxygenator obstruction led to a measurable increase in shunt flow. This suggests that flow monitoring could be a promising approach to identifying obstructions, offering an alternative to pressure-based monitoring [26].

These studies underscore the importance of monitoring flow and pressures within the oxygenator to detect dysfunction and clot formation during ECMO therapy. Flow monitoring shows promise as a non-contact and continuous method to identify obstructions in the circuit. Effective monitoring strategies and early recognition of ML dysfunction can guide decisions on the need for oxygenator replacement, ensuring optimal ECMO performance and patient safety. Further research and validation of flow monitoring as a reliable indicator are needed to enhance ECMO management.

### Anticoagulation strategy

Achieving the appropriate balance of anticoagulation in extracorporeal membrane oxygenation (ECMO) therapy is crucial, but challenging, as excessive anticoagulation can lead to bleeding complications. Several studies have examined the role of anticoagulation in ECMO and the associated management difficulties.

ECMO poses challenges in maintaining proper hemostasis due to blood’s interaction with artificial surfaces, leading to a pro-thrombotic state. Complications, such as bleeding and thrombosis, are common. Anticoagulation is vital to balance these risks, with unfractionated heparin (UFH) being the primary choice, but it has limitations and requires close monitoring using tests like ACT and aPTT. Acquired antithrombin deficiency is common and needs supplementation. Direct thrombin inhibitors (DTIs) like bivalirudin are alternatives but require careful dosing. Anticoagulation protocols in ECMO vary, underlining the need for individualized approaches and multidisciplinary teams to optimize patient care. The study lacks original clinical data and offers limited insights into alternative anticoagulants, fibrinolysis, and anticoagulation monitoring. It acknowledges heterogeneity in anticoagulation practices but provides no specific solutions. Overall, while informative, the study would benefit from additional research and data to address these limitations and offer practical recommendations for ECMO management [1].

A systematic review by McBane II et al. focuses on the thrombosis risk, prognostic implications, and anticoagulation effects in COVID-19 patients. The review highlights the high incidence of coagulopathy and thrombotic events in severe COVID-19 cases, emphasizing the importance of anticoagulation in these individuals [27].

Chlebowski et al. discuss the challenges associated with anticoagulation management during ECMO, which can result in high rates of both thrombotic and bleeding events. Factors such as age-related changes in hemostasis and blood-circuit interaction contribute to the difficulty of achieving optimal anticoagulation. Heparin resistance, characterized by the need for increasing heparin doses, is a common issue during ECMO, particularly in neonatal and pediatric patients with lower antithrombin (AT) levels. The controversy surrounding AT supplementation for heparin resistance highlights the need for clearer guidelines. The study reveals significant practice variation among medical centers due to a lack of standardized protocols and high-quality data. To optimize anticoagulation in ECMO, the study suggests considering multiple measures of hemostasis and emphasizes the importance of monitoring antithrombin levels. Developing more precise hemostasis assessment tools is essential for improving anticoagulation management in this complex clinical setting [28].

Studies have also examined the association between anticoagulation therapy and clinical outcomes in ECMO patients. Song et al. found that maintaining a higher activated partial thromboplastin time (aPTT) increased the risk of bleeding events without significantly improving thrombotic outcomes. This highlights the delicate balance required in anticoagulation management during ECMO [29].

Managing anticoagulation often involves the need for an oxygenator or circuit change out in cases of significant clot formation or dysfunction. Case reports have highlighted the importance of prompt intervention and alternative anticoagulants in restoring gas exchange and optimizing ECMO performance [30].

Furthermore, monitoring anti-Factor Xa levels has shown promise in assessing coagulation status and predicting the need for circuit/membrane oxygenator change in pediatric ECMO patients [2].

Direct thrombin inhibitors (DTIs) have been a good alternative to heparin anticoagulation in recent years, minimizing heparin-associated complications like acquired antithrombin deficiency and heparin-induced thrombocytopenia. Burstein et al. explain the safe usage of DTIs in ECMO due to their relatively short half-life, and ease of monitoring using activated partial thromboplastin time (aPTT) or activated clotting time (ACT). Dosing and monitoring protocols should be in place due to possible complications, for example, using Bivalirudin with low flow states such as weaning off ECMO or low pulsatility states may have to be supplemented with heparin due to Bivalirudin being rapidly cleaved by proteolytic enzymes [31]. M’pembele et al also showed beneficial effects on clinical outcomes in ECMO patients using DTIs as compared to heparin [32]. Geli et al. is another study showing DTIs to be a viable alternative, especially in HITT and heparin resistance patients such as COVID-19 patients [33], and Neunert et al. showed DTI use in pediatric patients safely, highlighting the potential to avoid use of antithrombin replacement and reduce lab monitoring [34].

In summary, anticoagulation is crucial in ECMO to prevent clot formation and maintain optimal oxygenator function. Balancing anticoagulation therapy poses challenges, and standardized guidelines and high-quality data are needed. An oxygenator or circuit change-out may be necessary in cases of significant clot formation or dysfunction. Advancements in anticoagulation management during ECMO are necessary to improve patient outcomes.

### Key considerations for performing ECMO circuit change out

**Figure 1:**
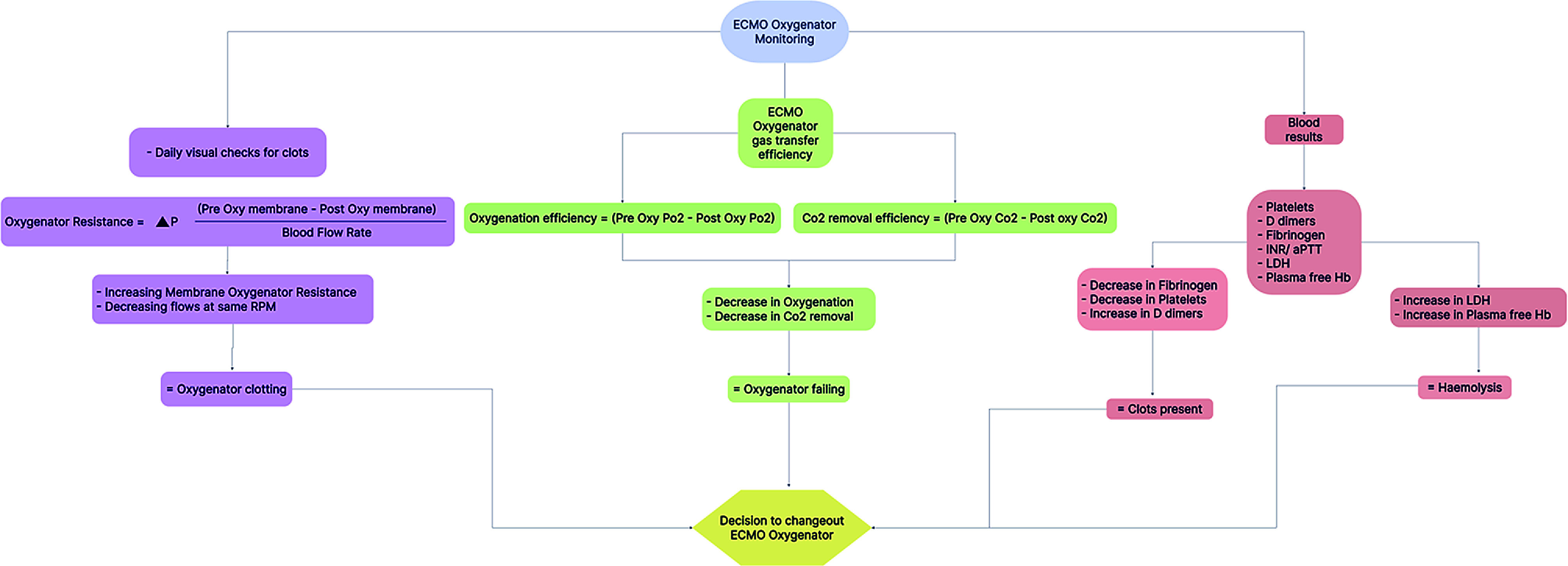
Flow chart A: Illustration of Factors to be considered before ECMO circuit/oxygenator change-out.

Changing an ECMO circuit is a critical procedure that requires careful technique, thorough preparation, and consideration of various factors (Figure 1):

- *Technique*: The procedure should be performed with strict adherence to aseptic technique to minimize the risk of infections. Proper technique and standard operating procedure are essential to ensure the safe and efficient exchange of the circuit.
- *Preparation*: Adequate preparation is crucial, including ensuring the availability of a fully primed and functional replacement circuit, appropriate anticoagulation management, and proper monitoring equipment. Clear communication among the multidisciplinary team involved in the procedure is essential for a smooth transition.
- *Patient stability*: The patient’s hemodynamic stability, oxygenation, and coagulation status should be carefully assessed before the circuit change. It is crucial to optimize the patient’s condition to minimize the risk of complications during the procedure.
- *Timelines*: Determining the optimal timing for circuit change-out is essential and should be based on the patient’s stability and minimized risk of complications. Close coordination with the ECMO specialist team and careful evaluation of the patient’s clinical status are crucial factors in identifying the ideal time to perform the circuit exchange. By considering these aspects, clinicians can ensure a safe and effective procedure, maximizing the chances of successful patient outcomes.
- *Post-procedure monitoring*: Close monitoring of the patient’s vital signs, oxygenation, and coagulation parameters should be continued after the circuit change. Assessing the circuit function, adequate blood flow, and gas exchange is essential to ensure the patient’s stability and the proper functioning of the new ECMO circuit.

Siddiqui et al. propose a staged approach for circuit exchanges in fully ECLS-dependent patients to avoid hemodynamic deterioration. By gradually transitioning flow from the old to the new circuit in a parallel configuration, the staged approach ensures hemodynamic stability even in patients without native organ function, reducing the risks associated with circuit exchanges [35].

Da Broi et al. evaluated a new system and procedure for oxygenator change-out in ECC and ECMO. The study demonstrated that the new system allowed for a fast and efficient change-out process, completed in a short average time. This new system offers a safe and reproducible method for oxygenator change-out, minimizing potential risks associated with the procedure [36].

**Figure 2:**
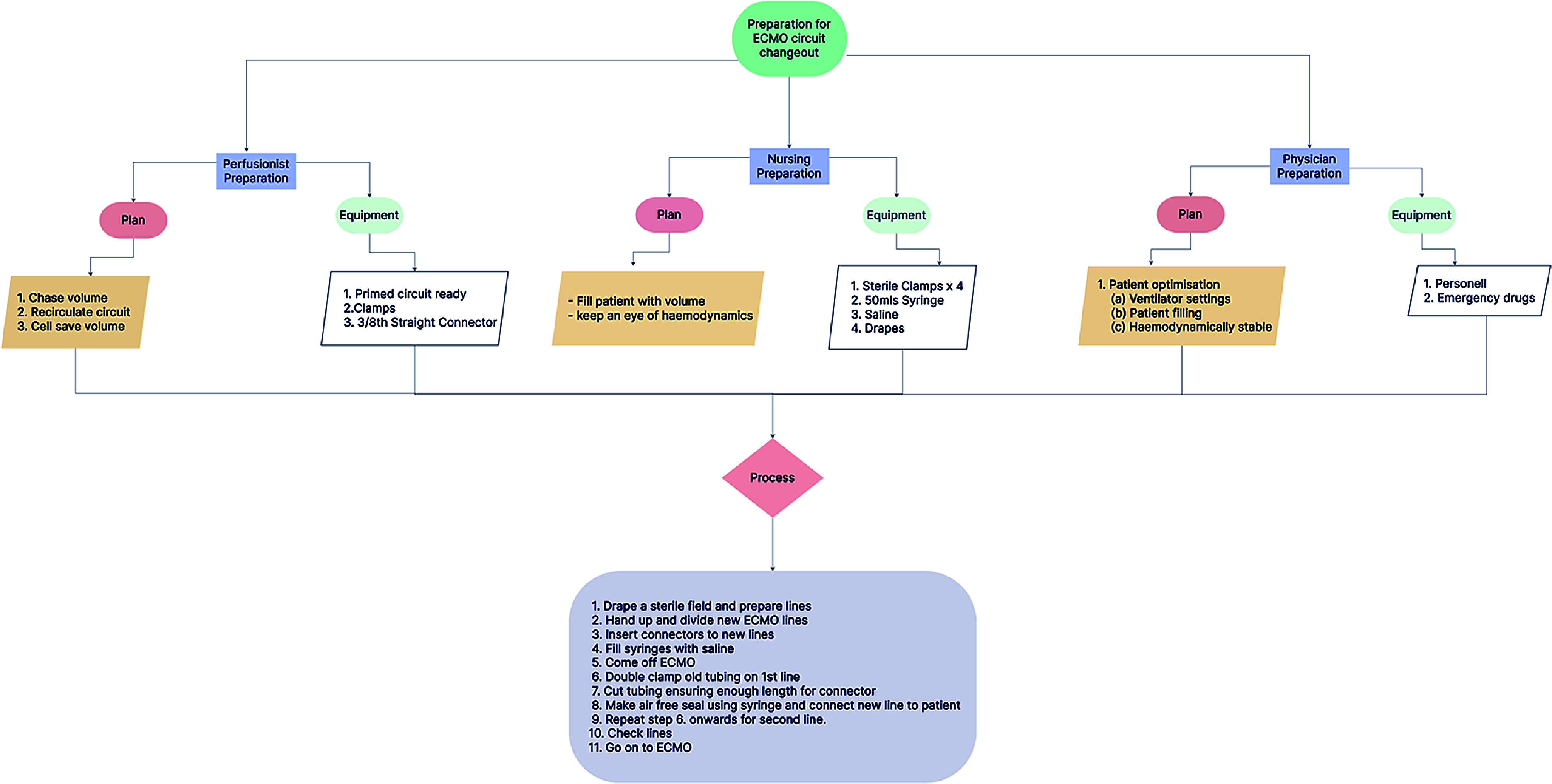
Flow Chart B: Illustration of standard operating procedure of ECMO circuit change-out.

In summary, established protocols and guidelines can help ensure a successful ECMO circuit change with minimized risks to the patient (Figure 2).

### ELSO guidelines

The ELSO guidelines on anticoagulation and circuit changeout provide comprehensive recommendations for the management of anticoagulation therapy and the timely replacement of oxygenators in patients undergoing extracorporeal membrane oxygenation (ECMO). The guidelines emphasize the importance of achieving a delicate balance between preventing thrombosis and minimizing bleeding complications. They recommend the use of unfractionated heparin as the primary anticoagulant, with regular monitoring of coagulation parameters such as activated clotting time (ACT), activated partial thromboplastin time (aPTT), and anti-Xa levels. The guidelines also highlight the importance of closely monitoring oxygenator performance and replacing it when necessary, based on established criteria such as clot formation, compromised blood flow, and gas exchange impairment. By providing evidence-based recommendations, these guidelines aim to optimize anticoagulation management and ensure appropriate maintenance of the oxygenator, ultimately improving patient outcomes during ECMO therapy [37].

## Future considerations for oxygenator or circuit change-out

While current literature provides valuable insights into the considerations for oxygenator or circuit change-out in patients undergoing extracorporeal membrane oxygenation (ECMO), several areas warrant further research and exploration. Addressing these future considerations and knowledge gaps can enhance our understanding and improve the decision-making process for change-outs.

- *Personalized anticoagulation strategies*: Currently, anticoagulation therapy is often administered empirically based on general guidelines. However, individual patient factors such as age, comorbidities, and coagulation profile may influence the response to anticoagulants and the risk of bleeding. Future research should focus on developing personalized anticoagulation strategies that take into account patient-specific characteristics, allowing for tailored therapy to achieve optimal circuit patency while minimizing bleeding complications.
- Although conventional coagulation parameters like ACT, aPTT, and anti-Xa levels provide valuable information, their ability to predict clotting or bleeding risks is limited. Therefore, there is a need to identify novel biomarkers that can better reflect the coagulation status and accurately predict the likelihood of circuit clotting or bleeding complications. Exploring biomarkers associated with platelet function, endothelial activation, or fibrinolysis could provide additional insights and significantly improve the monitoring and management of anticoagulation in ECMO patients. By incorporating these novel biomarkers, clinicians can make more informed decisions and optimize anticoagulation strategies for better patient outcomes.
- *Point of care test*: POC tests play a crucial role in ensuring patients are within the therapeutic range of antithrombotic medications, identifying individuals at risk for bleeding or thrombotic complications, and guiding the management of acute bleeding episodes. Nonetheless, there is a pressing need for additional research to establish clinically relevant ranges for POC coagulation testing in patients on VADs and ECMO circuits. Anticoagulation strategies vary between these two patient populations, with unfractionated heparin being the primary choice for ECMO, and antiplatelet medications sometimes used in combination with heparin for VAD patients. The correlation between traditional coagulation tests and POC tests like TEG and ROTEM is inconsistent, and no clear superiority of POC tests over standard tests has been established in terms of monitoring anticoagulation or predicting bleeding or thrombotic complications. Thus, further research is essential to validate the predictive ranges of POC tests and develop reliable and accurate POC versions of standard coagulation tests.
- *Non-invasive monitoring techniques*: Current monitoring of coagulation parameters typically involves invasive blood sampling, which may not be ideal for continuous monitoring in critically ill patients. Developing non-invasive techniques for real-time assessment of coagulation status, such as point-of-care devices or wearable sensors, could revolutionize anticoagulation management in ECMO. These technologies could provide immediate feedback on coagulation status, allowing for timely interventions and adjustments to anticoagulation therapy.
- *Optimal timing for change-out*: The timing of the oxygenator or circuit change-out remains a topic of debate. There is limited evidence on the ideal time to perform a change-out to minimize complications and optimize patient outcomes. Future research should focus on identifying new indicators that can predict the need for change-out, such as biomarkers, imaging modalities, or clinical parameters. Determining the optimal timing for change-out can help prevent circuit clotting and reduce the risk of complications.
- Safe and efficient ECMO circuit change-out requires thorough preparation, collaboration, and the development of standard operating procedures (SOPs) among doctors, nurses, and perfusionists. SOPs provide clear guidelines and protocols to ensure consistent and standardized practices, enhancing patient safety and procedural efficiency. Effective teamwork, communication, and adherence to established SOPs are essential for successful circuit change-outs in ECMO therapy.
- *Long-term outcomes and complications*: Most studies and guidelines focus on short-term outcomes and complications related to oxygenator or circuit change-out. However, there is a need for long-term follow-up studies to assess the impact of change-outs on patient survival, organ function, and quality of life. Understanding the long-term consequences and potential complications associated with change-outs can guide decision-making and improve patient counseling and care [25, 36, 37, 38, 39, 40].

## Conclusion

In conclusion, this review sheds light on the pivotal role of extracorporeal membrane oxygenation (ECMO) in critical care, particularly for patients suffering from severe cardiac or pulmonary dysfunction, including acute respiratory distress syndrome (ARDS) and critical COVID-19 cases.

It underscores the crucial importance of maintaining ECMO’s performance through timely component replacements, with a primary focus on the oxygenator, to ensure the therapy’s continued effectiveness and safety. The paper provides a thorough analysis of the key factors influencing the decision to change ECMO components, with a particular emphasis on monitoring parameters such as fibrinogen levels, pre and post-membrane blood gases, plasma free hemoglobin, D-dimers, platelet levels, flows, pressures, and anticoagulation strategies. While individually they are not a direct indication to change the ECMO circuit, but collectively they can provide a holistic picture of the workings of the ECMO. These factors are essential in identifying and addressing complications like bleeding and thrombosis, which can significantly impact patient outcomes.

Furthermore, the review not only highlights the current state of knowledge and best practices but also outlines future considerations for ECMO component change-outs. These considerations encompass the development of personalized anticoagulation strategies, exploration of novel biomarkers, validation of point-of-care tests, the advancement of non-invasive monitoring techniques, determination of optimal timing for change-outs, the establishment of standardized operating procedures, and long-term outcome assessments.

In summary, this review provides valuable insights for healthcare professionals and researchers, offering a comprehensive overview of ECMO therapy and guiding future directions in the field. It emphasizes the ongoing commitment to enhancing patient care, improving ECMO management, and ultimately saving lives in critical care scenarios.

## Data Availability

The research data are available on request from the authors.

## References

[1] Kumar G, Maskey A. Anticoagulation in ECMO patients: an overview. Indian J Thorac Cardiovasc Surg. 2021;37:241–247. 33967447 10.1007/s12055-021-01176-3 PMC8062644

[2] Irby K, Swearingen C, Byrnes J, Bryant J, Prodhan P, Fiser R. Unfractionated heparin activity measured by anti-factor Xa levels is associated with the need for extracorporeal membrane oxygenation circuit/membrane oxygenator change: a retrospective pediatric study. Pediatr Crit Care Med. 2014;15(4):e175–e182. 24622165 10.1097/PCC.0000000000000101 PMC4013211

[3] Genty T, Burguburu S, Imbert A, Roman C, Camille W, Thès J, Stéphan F. Circuit change during extracorporeal membrane oxygenation: single-center retrospective study of 48 changes. Critical Care. 2023;27(1):1–6. 37269022 10.1186/s13054-023-04503-9 PMC10239194

[4] Lubnow M, Philipp A, Dornia C, et al. D-dimers as an early marker for oxygenator exchange in extracorporeal membrane oxygenation. J Crit Care. 2014;29(3):473–e1. 10.1016/j.jcrc.2013.12.008 24508200

[5] Basken R, Cosgrove R, Malo J, et al. Predictors of oxygenator exchange in patients receiving extracorporeal membrane oxygenation. J Extra Corpor Technol. 2019;51(2):61. 31239577 PMC6586268

[6] Worku ET, Win AM, Parmar D, Anstey C, Shekar K. Haematological trends and transfusion during adult extracorporeal membrane oxygenation: a single centre study. J Clin Med. 2023;12(7):2629. 37048711 10.3390/jcm12072629 PMC10095131

[7] Doyle AJ, Hunt BJ. Current understanding of how extracorporeal membrane oxygenators activate haemostasis and other blood components. Front Med. 2018;12(5):352. 10.3389/fmed.2018.00352 PMC6299009 30619862

[8] Mansour A, Flecher E, Schmidt M, et al. Bleeding and thrombotic events in patients with severe COVID-19 supported with extracorporeal membrane oxygenation: a nationwide cohort study. Intensive Care Med. 2022;48(8):1039–1052. 35829723 10.1007/s00134-022-06794-y

[9] Epis F, Belliato M. Oxygenator performance and artificial-native lung interaction. J Thorac Dis. 2018;10(Suppl 5):S596. 29732176 10.21037/jtd.2017.10.05 PMC5911558

[10] Belliato M, Degani A, Buffa A, et al. A brief clinical case of monitoring of oxygenator performance and patient-machine interdependency during prolonged veno-venous extracorporeal membrane oxygenation. J Clin Monit Comput. 2017;31:1027–1033. 27558734 10.1007/s10877-016-9927-4

[11] Dufour N, Radjou A, Thuong M. Hemolysis and plasma free hemoglobin during extracorporeal membrane oxygenation support: from clinical implications to laboratory details. ASAIO J. 2020;66(3):239–246. 30985331 10.1097/MAT.0000000000000974

[12] Omar HR, Mirsaeidi M, Socias S, et al. Plasma free hemoglobin is an independent predictor of mortality among patients on extracorporeal membrane oxygenation support. PLoS One. 2015;10(4), e0124034. 25902047 10.1371/journal.pone.0124034 PMC4406730

[13] Pan KC, McKenzie DP, Pellegrino V, Murphy D, Butt W. The meaning of a high plasma free haemoglobin: retrospective review of the prevalence of haemolysis and circuit thrombosis in an adult ECMO centre over 5 years. Perfusion. 2016;31(3):223–231. 26201941 10.1177/0267659115595282

[14] Valladolid C, Yee A, Cruz MA. von Willebrand factor, free hemoglobin and thrombosis in ECMO. Front Med. 2018;17(5):228. 10.3389/fmed.2018.00228 PMC6107708 30175099

[15] Hazboun RG, Darwish N, Rotyliano-Sykes G, et al. Predictors of circuit health in neonatal patients receiving extracorporeal membrane oxygenation (ECMO). Sci Rep. 2022;12(1):1265. 35075252 10.1038/s41598-022-05389-3 PMC8786946

[16] Neal JR, Quintana E, Pike RB, Hoyer JD, Joyce LD, Schears G. Using daily plasma-free hemoglobin levels for diagnosis of critical pump thrombus in patients undergoing ECMO or VAD support. J Extra Corpor Technol. 2015;47(2):103. 26405358 PMC4557546

[17] Appelt H, Philipp A, Mueller T, et al. Factors associated with hemolysis during extracorporeal membrane oxygenation (ECMO) – Comparison of VA – versus VV ECMO. PLOS ONE [Internet]. 2020;15(1):e0227793. 31986168 10.1371/journal.pone.0227793 PMC6984694

[18] Dornia C, Philipp A, Bauer S, et al. D-dimers are a predictor of clot volume inside membrane oxygenators during extracorporeal membrane oxygenation. Artif Organs. 2015;39(9):782–787. 25845704 10.1111/aor.12460

[19] Shakoor A, Chen S, Hyde J, et al. Utility of D-dimers in COVID-19 patients requiring extracorporeal membrane oxygenation. ASAIO J. 2022;68(10):1241. 35609187 10.1097/MAT.0000000000001775 PMC9521394

[20] Balle CM, Jeppesen AN, Christensen S, Hvas AM. Platelet function during extracorporeal membrane oxygenation in adult patients. Front Cardiovasc Med. 2019;8(6):114. 10.3389/fcvm.2019.00114 PMC6694790 31440518

[21] Chegondi M, Vijayakumar N, Badheka A, Karam O. Effect of platelet transfusions on extracorporeal life support oxygenator’s function. Front Pediatr. 2022;7(10):826477. 10.3389/fped.2022.826477 PMC8936087 35321010

[22] Balle CM, Jeppesen AN, Christensen S, Hvas AM. Platelet function during extracorporeal membrane oxygenation in adult patients: a systematic review. Front Cardiovasc Med. 2018;9(5):157. 10.3389/fcvm.2018.00157 PMC6237979 30474031

[23] Winnersbach P, Rossaint J, Buhl EM, et al. Platelet count reduction during in vitro membrane oxygenation affects platelet activation, neutrophil extracellular trap formation and clot stability, but does not prevent clotting. Perfusion. 2022;37(2):134–143. 33475044 10.1177/0267659121989231 PMC8928426

[24] Steiger T, Foltan M, Philipp A, et al. Accumulations of von Willebrand factor within ECMO oxygenators: Potential indicator of coagulation abnormalities in critically ill patients? Artif Organs. 2019;43(11):1065–1076. 31192471 10.1111/aor.13513 PMC6899554

[25] Zakhary B, Vercaemst L, Mason P, Antonini MV, Lorusso R, Brodie D. How I approach membrane lung dysfunction in patients receiving ECMO. Crit Care. 2020;24:1–4. 33256824 10.1186/s13054-020-03388-2 PMC7704102

[26] Sarathy S, Turek JW, Chu J, Badheka A, Nino MA, Raghavan ML. Flow monitoring of ECMO circuit for detecting oxygenator obstructions. Ann Biomed Eng. 2021;49:3636–3646. 34705123 10.1007/s10439-021-02878-w

[27] McBane RD II, Roldan VD, Niven AS, et al. Anticoagulation in COVID-19: a systematic review, meta-analysis, and rapid guidance from Mayo Clinic. Mayo Clinic Proc. 2020;95(11):2467–2486. Elsevier. 10.1016/j.mayocp.2020.08.030 PMC7458092 33153635

[28] Chlebowski MM, Baltagi S, Carlson M, Levy JH, Spinella PC. Clinical controversies in anticoagulation monitoring and antithrombin supplementation for ECMO. Critical Care 2020;24:1–2. 31959232 10.1186/s13054-020-2726-9 PMC6971875

[29] Song K, Kim JB. Safety of low-dose anticoagulation in extracorporeal membrane oxygenation using the Permanent Life Support System: a retrospective observational study. J Yeungnam Med Sci 2023;40(3):276–282. 37198890 10.12701/jyms.2023.00339 PMC10390269

[30] Ratzlaff RA, Ripoll JG, Kassab LL, Diaz-Gomez JL. Acute oxygenator failure: a new presentation of heparin-induced thrombocytopenia in a patient undergoing venovenous extracorporeal membrane oxygenation support. Case Rep. 2016;16(2016):bcr2016218179. 10.1136/bcr-2016-218179 PMC5174850 27986695

[31] Burstein B, Wieruszewski PM, Zhao YJ, Smischney N. Anticoagulation with direct thrombin inhibitors during extracorporeal membrane oxygenation. World J Crit Care Med. 2019;8(6):87. 31750086 10.5492/wjccm.v8.i6.87 PMC6854393

[32] M’Pembele R, Roth S, Metzger A, et al. Evaluation of clinical outcomes in patients treated with heparin or direct thrombin inhibitors during extracorporeal membrane oxygenation: a systematic review and meta-analysis. Thrombosis J. 2022;20(1):1–9. 10.1186/s12959-022-00401-2 PMC9330661 35902857

[33] Geli J, Capoccia M, Maybauer DM, Maybauer MO. Direct thrombin inhibition in extracorporeal membrane oxygenation. Int J Artif Organs. 2022;45(7):652–655. 35411823 10.1177/03913988221091292

[34] Neunert C, Chitlur M, Van Ommen CH. The changing landscape of anticoagulation in pediatric extracorporeal membrane oxygenation: use of the direct thrombin inhibitors. Front Med. 2022;6(9):887199. 10.3389/fmed.2022.887199 PMC9299072 35872781

[35] Siddiqui NA, Dominguez A, Sharma A, Conrad SA. Technique for circuit exchange in high-risk patients on extracorporeal life support. Perfusion 2023;38(5):963–5. 35514054 10.1177/02676591221096223 PMC10265286

[36] Da Broi U, Adami V, Falasca E, Malangone W, Crini S, Degrassi A. A new oxygenator change-out system and procedure. Perfusion. 2006;21(5):297–303. 17201085 10.1177/0267659106074771

[37] McMichael AB, Ryerson LM, Ratano D, Fan E, Faraoni D, Annich GM. 2021 ELSO adult and pediatric anticoagulation guidelines. ASAIO J. 2022;68(3):303–310. 35080509 10.1097/MAT.0000000000001652

[38] Zeibi Shirejini S, Carberry J, McQuilten ZK, Burrell AJ, Gregory SD, Hagemeyer CE, Current and future strategies to monitor and manage coagulation in ECMO patients. Thrombosis J. 2023;21(1):1–20. 10.1186/s12959-023-00452-z PMC9878987 36703184

[39] Delmas C, Jacquemin A, Vardon-Bounes F, et al. Anticoagulation monitoring under ECMO support: a comparative study between the activated coagulation time and the anti-Xa activity assay. J Intensive Care Med. 2020;35(7):679–686. 29768983 10.1177/0885066618776937

[40] Bercovitz RS. An introduction to point-of-care testing in extracorporeal circulation and LVADs. Hematology 2018;2018(1):516–521. 30504352 10.1182/asheducation-2018.1.516 PMC6245959

